# Age-related Aortic Stiffness Can Be Transferred and Ameliorated via Fecal Microbiota Transplant in Mice.

**DOI:** 10.1093/geroni/igab046.3022

**Published:** 2021-12-17

**Authors:** Nathan Greenberg, Nicholas VanDongen, Rachel Gioscia-Ryan, Abigail Casso, David Hutton, Zachary Clayton, Douglas Seals, Vienna Brunt

**Affiliations:** 1 University of Colorado at Boulder, Boulder, Colorado, United States; 2 University of Colorado Boulder, Boulder, Colorado, United States

## Abstract

Age-related increases in aortic stiffness contribute to the development of cardiovascular diseases (CVD). To determine whether the gut microbiome (GM) modulates age-related aortic stiffening, we performed fecal microbiota transplants (FMT) between young (Y; 3 month) and older (O; 25 month) male C57BL/6N mice. Following antibiotic treatment (to suppress endogenous microbiota), mice received weekly FMT (fecal samples collected at baseline) via oral gavage for 8-16 weeks from their own (i.e., sham condition: Y-y, O-o [RECIPIENT-donor]) or opposite age group (Y-o, O-y) (N=8-12/group). In vivo aortic stiffness (pulse wave velocity [PWV]) was higher in older vs. young mice at baseline (382±8 vs. 328±7cm/sec, mean±SE, P<0.001). Arterial phenotypes were transferred such that old microbiota transplanted into young mice increased, while young into old decreased, PWV (Y-y: 325±10 vs. Y-o: 362±10cm/sec, P=0.022; O-o: 409±10 vs. O-y: 335±6cm/sec, P<0.001). Intrinsic mechanical stiffness of excised aortic rings (elastic modulus) increased after transplant of old into young (Y-y: 2141±223 vs. Y-o: 3218±394kPA, P=0.022), and decreased with young into old (O-O: 3263±217 vs. O-y: 2602±136kPA, P=0.016), indicating the GM mediates aortic stiffening by modulating structural changes in the arterial wall. Age-related increases in aortic abundance of advanced glycation end products (AGEs), which cross-link arterial structural proteins, tended to be transferred by the GM (Y-y: 0.022±0.001 vs. Y-o: 0.038±0.006 A.U., P=0.11; O-o: 0.120±0.029 vs. O-y: 0.038±0.009 A.U., P=0.06). The aging GM can induce aortic stiffening via promoting AGEs accumulation and crosslinking of arterial structural proteins, and thus might be a promising target for preventing/treating age-related aortic stiffening and CVD.

